# Quantitative observation of monochromatic X-rays emitted from implosion hotspot in high spatial resolution in inertial confinement fusion

**DOI:** 10.1038/s41598-021-93482-4

**Published:** 2021-07-14

**Authors:** Kuan Ren, Junfeng Wu, Jianjun Dong, Yaran Li, Tianxuan Huang, Hang Zhao, Yaoyuan Liu, Zhurong Cao, Jiyan Zhang, Baozhong Mu, Ji Yan, Wei Jiang, Yudong Pu, Yulong Li, Xiaoshi Peng, Tao Xu, Jiamin Yang, Ke Lan, Yongkun Ding, Shaoen Jiang, Feng Wang

**Affiliations:** 1 Research Center of Laser Fusion, China Academy of Engineering Physics, Mianyang, 621900 China; 2 Institute of Applied Physics and Computational Mathematics, Beijing, 100088 China; 3 MOE Key Laboratory of Advanced Micro-Structured Materials, School of Physics Science and Engineering, Tongji University, 1239 Siping Rd., Shanghai, 200092 China; 4 CAS Key Laboratory of Geospace Environment and Department of Engineering and Applied Physics, University of Science and Technology of China, Hefei, 230027 Anhui China; 5 CAPT, HEDPS, and IFSA Collaborative Innovation Center of MoE, Peking University, Beijing, 100871 China

**Keywords:** Nuclear fusion and fission, Plasma physics, Laser-produced plasmas

## Abstract

In inertial confinement fusion, quantitative and high-spatial resolution ($< 10\,\upmu$m) measurements of the X-rays self-emitted by the hotspot are critical for studying the physical processes of the implosion stagnation stage. Herein, the 8 ± 0.39-keV monochromatic X-ray distribution from the entire hotspot is quantitatively observed in 5-$\upmu$m spatial resolution using a Kirkpatrick–Baez microscope, with impacts from the responses of the diagnosis system removed, for the first time, in implosion experiments at the 100 kJ laser facility in China. Two-dimensional calculations along with 2.5% P2 drive asymmetry and 0.3 ablator self-emission are congruent with the experimental results, especially for the photon number distribution, hotspot profile, and neutron yield. Theoretical calculations enabled a better understanding of the experimental results. Furthermore, the origins of the 17.81% contour profile of the deuterium-deuterium hotspot and the accurate Gaussian source approximation of the core emission area in the implosion capsule are clarified in detail. This work is significant for quantitatively exploring the physical conditions of the hotspot and updating the theoretical model of capsule implosion.

## Introduction

Quantitative and high-space-resolving measurements of the physical conditions of the hotspot self-emitting area, which removes the impacts from the response of the diagnosis system, forms the foundation for further research on relative physical processes of the implosion stagnation stage in inertial confinement fusion (ICF)^1,2^. For example, small variations in the radius of the hotspot will lead to a large change in the neutron yield of the ignition capsule ($Y\sim p^{2}T^{2}R^{3}_{hs}\tau _{bw}$, where *Y* is the neutron yield, $R_{hs}$ is the radius of the hotspot, *p* is the pressure, *T* is the temperature, and $\tau _{bw}$ is the burn-width)^3^.

Conventional quantitative detections of hotspot self-emission use a pinhole imaging technique^1,4,5^. However, this technique has a relatively low spatial resolution ($\ge$ 10 $\upmu$m) for hotspots of 30–60 $\upmu$m^6,7^. Many hotspots have been observed through high-space-resolving observations (< 10 $\upmu$m) in implosion experiments using a Kirkpatrick–Baez (KB) microscope^8–11^. Considering the errors in the aiming of the KB microscope, it is difficult to relate the X-ray image to the reflectivity distribution of the microscope in a pixel to pixel manner. Consequently, removing the impact of the diagnosis system response (the reflectivity distribution) in the recorded X-ray image data is also difficult; that impact even changes the hotspot profile and distribution. Thus, quantitative measurements are difficult to obtain. Although quantitative detection of X-ray emission for hotspot doping elements such as Kr^12^ and Ar^13^ is widely used to investigate stagnation stage physics, the radiation cooling effect introduced by the doping elements in the hotspot cannot be ignored^14^.

In this work, we solve the quantization problem by using the published simulation method of reflectivity distribution of a KB microscope^15^ that has an aiming error of less than ± 20 $\upmu$m, and then placing the uncertainty caused by the corresponding error that occurs while relating an X-ray image to reflectivity distributions of the KB microscope, in a pixel to pixel manner, into the final data uncertainties. Thus, the 8 ± 0.39-keV monochromatic X-rays emitted by the hotspot were quantitatively measured with 5-$\upmu$m spatial resolution using an eight-image KB microscope^15^ and, for the first time, without the impacts from the diagnosis system response. This was achieved in implosion experiments performed at the 100 kJ laser facility of China^16,17^. Several characteristics of the hotspot, such as the number distributions of the monochromatic photon, geometrical profile, and dimension, were observed accurately. Theoretical simulation results are congruent with and further explain the experimental results. The P2 drive asymmetry could be 2.5%, and the self-emission from the ablator mixed into the hotspot is only 0.3 times the deuterium-deuterium hotspot emission, as obtained from the calculated results. We found that the calculated two-dimensional outline of the deuterium-deuterium hotspot is highly consistent with the 17.81% contour relative to the peak emission of the experimental results and that the core emission area of the experimental results could be accurately approximated as a Gaussian source. We conclude by discussing various uncertainties.

## Principles of KB microscope and preparation for quantization

**Figure 1:**
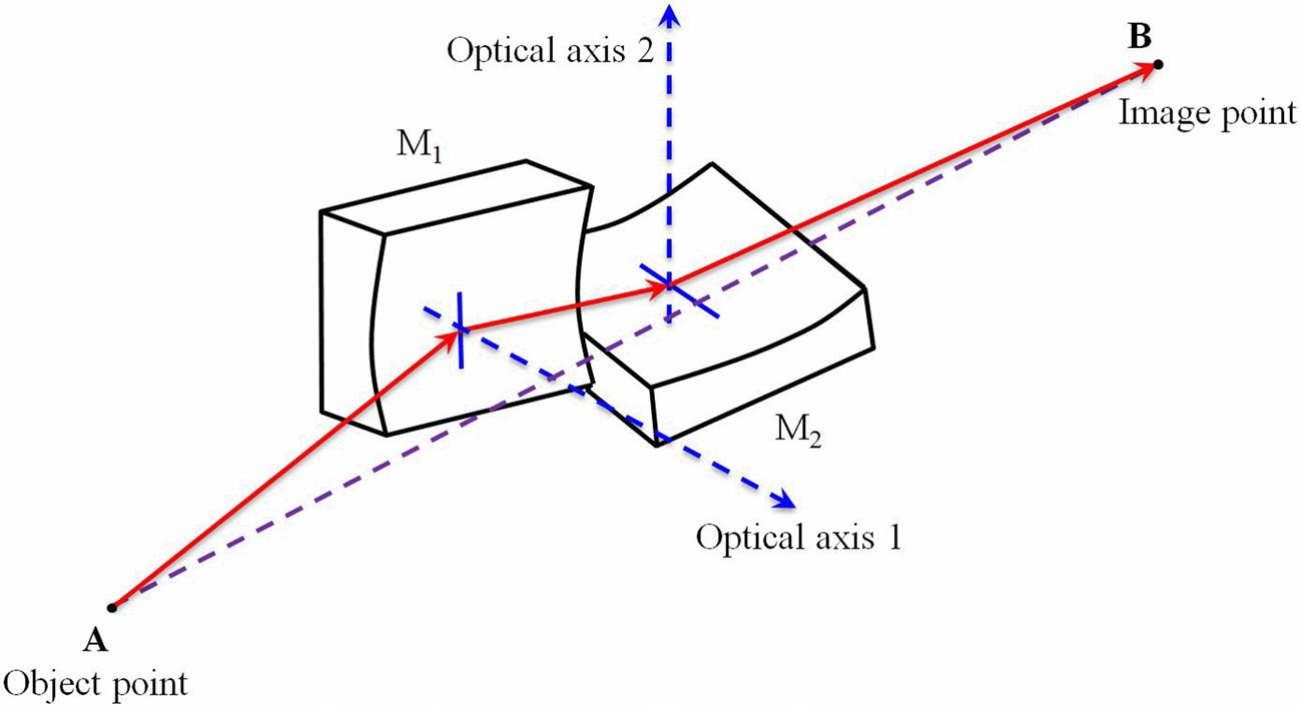
(Color online) Schematic illustration of KB microscope.

The KB microscope^8–11,15,18^ is commonly used for X-ray imaging and observation in ICF. Figure 1 shows the glancing incidence imaging principles of a KB microscope. This microscope consists of two spherical mirrors that are almost orthogonal, which results in a structure that can effectively decrease image aberration and improve spatial resolution, while also yielding similar spatial resolutions on the meridian and sagittal orientations. The mirror surface is coated with a multilayer film to improve the X-ray reflectivity. Together, the mirrors, film, and filters allow transmission of specific monochromatic light. Currently, the spatial resolution of a KB microscope can reach up to 2.5 $\upmu$m (the view field is approximately 100 $\upmu$m)^18^. The light-collection efficiency is 10–100 times higher than that of pinhole imaging, enabling direct observation of the relatively low hotspot self-emission. Further, because glancing incidence imaging and reflection away from the axis are used, the KB microscope can be easily shielded from stray light. In this work, the image plate was selected as the detector because it provides a large detection area and appropriate sensitivity and spatial resolution (better than 100 $\upmu$m^19,20^ and considering that the magnification of the KB microscope imaging is around 20 $\times$).

**Figure 2:**
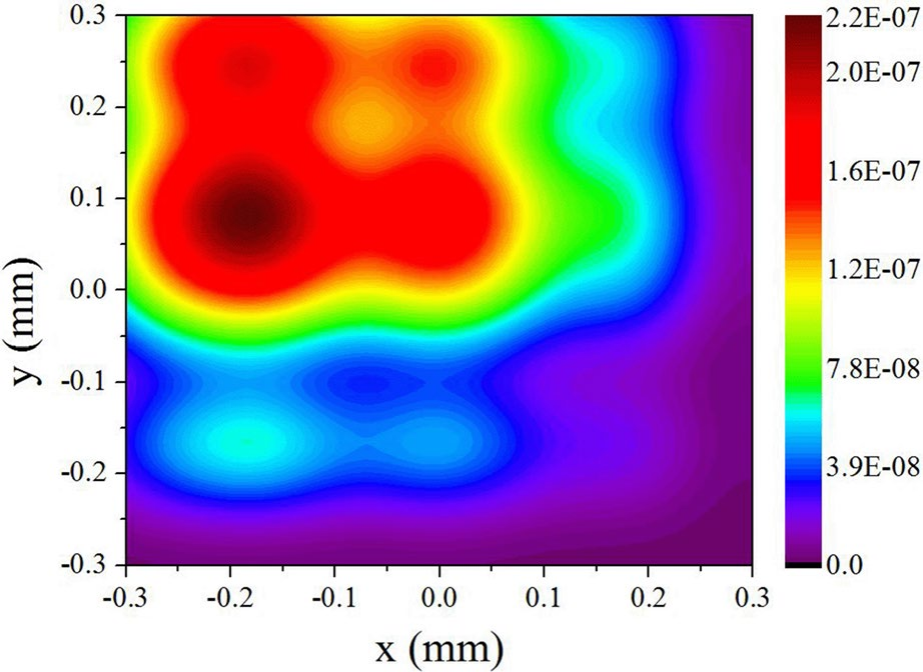
(Color online) System efficiency distribution map in the view field for the best channel of the eight-image KB microscope.

Through optimized design of the optics and structure, the eight-image KB microscope can simultaneously create eight X-ray images at different energy points from the same target^15^. Each of its channels is based on the front basic principle. In a recently published work, the reflectivity distribution $\eta$ (unit: sr) of one channel in the eight-image KB microscope was simulated and a high spatial resolution of 5 $\upmu$m in the view field range of ± 100 $\upmu$m was measured through backlit X-ray imaging of the mesh grid^15^. Here^15^,

1 $$\begin{aligned} {} \eta = \Omega \times R_1 \times R_2, \end{aligned}$$

where $\Omega$ (unit: sr) is the effective solid angle subtended by each KB microscope channel, and $R_1$ and $R_2$ are the reflectivities of the first and second spherical mirrors, respectively. Thus, the same simulation method was used to calculate the reflectivity distributions of all eight channels in the eight-image KB microscope. We chose the best channel, for which the reflectivity distribution $\eta$ varies less than 26.47% in any 20 $\upmu$m space range of ± 70 $\upmu$m scale, considering that the hotspot scale is less than ± 50 $\upmu$m and the error of aiming is less than ± 20 $\upmu$m, as shown in Fig. 2. This channel allows transmission of 8 ± 0.39-keV monochromatic X-rays. Thus, the absorption of those high-energy X-rays in the implosion capsule and the influence of the plasma in hohlraum can be ignored. In our work, the number distribution of the 8-keV monochromatic X-ray photons emitted from the hotspot is expressed as

2 $$\begin{aligned} {} N = \frac{4 \pi n}{\eta }, \end{aligned}$$

where *n* is the number distribution of the 8-keV monochromatic X-ray photons transmitted by the KB microscope, i.e., the recorded number of photons.

## Experiments and data process

**Figure 3:**
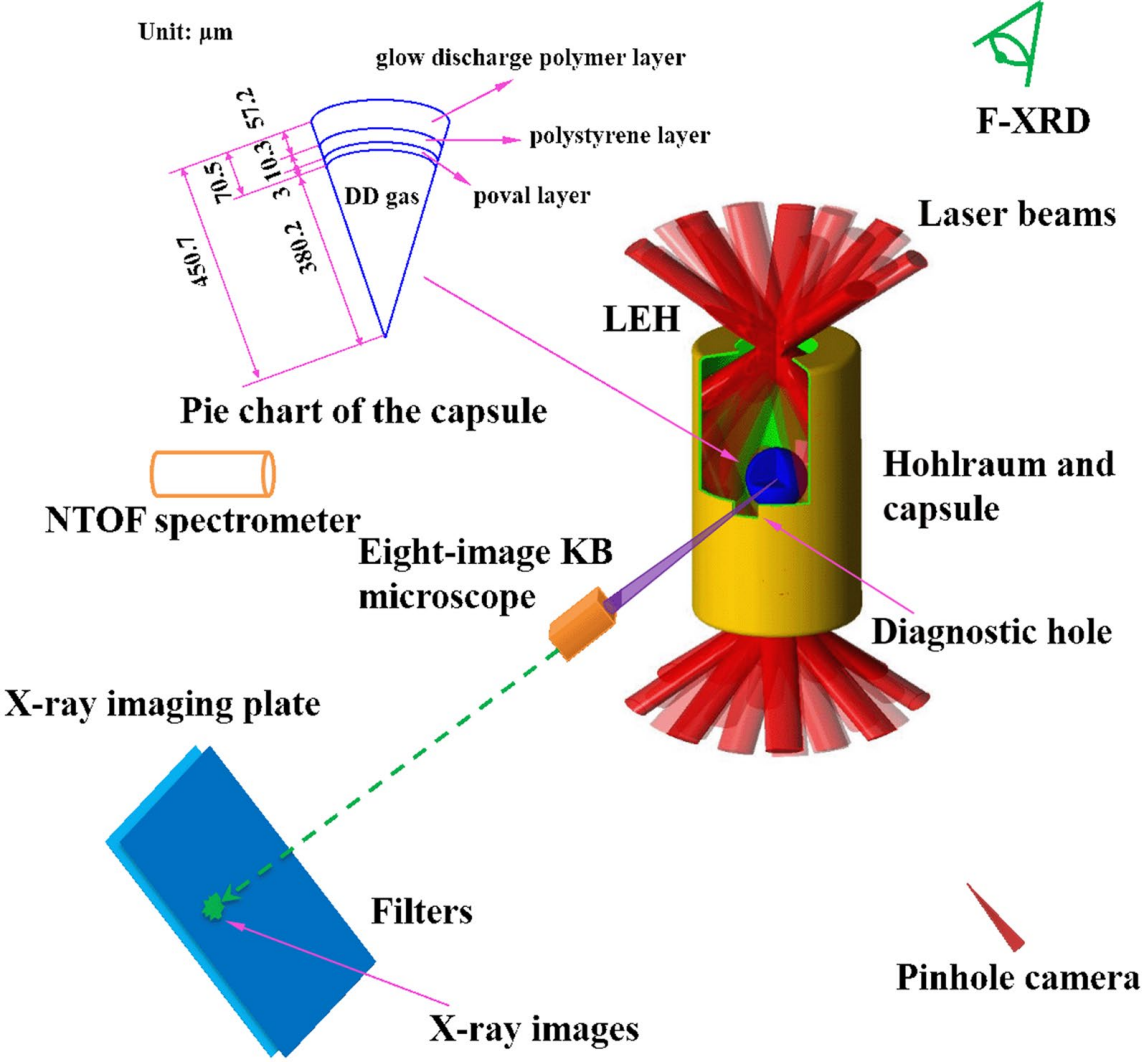
(Color online)Experimental setup for observation of hotspot self-emission.

Figure 3 shows the experimental setup. A 2400-$\upmu$m-diameter and 4000-$\upmu$m-long gas-filled hohlraum with two 1200–$\upmu$m–diameter laser entrance holes (LEHs) was used, at a pressure of 0.3 atm. Two 300 $\upmu$m $\times$ 300 $\upmu$m diagnostic holes were symmetrically dug on the hohlraum equator. A 901.4-$\upmu$m-diameter deuterium-deuterium (DD) capsule (DD gas with 10-atm pressure was filled in the center of a 760.4-$\upmu$m-diameter area that was surrounded by a 70.5-$\upmu$m-thick CH shell, which included a 3-$\upmu$m-thick and 1.3-g/cc-density poval (PVA) gas-captive layer, a 10.3-$\upmu$m-thick and 1.0-g/cc-density polystyrene (PS) supporting layer, and a 57.2-$\upmu$m-thick and 1%-Si-doping-concentration outer glow discharge polymer (GDP) layer) was positioned at the hohlraum center. Through the diagnostic hole, the DD capsule was viewed by the eight-image KB microscope. A Fuji BAS MS-type X-ray image plate (IP) and filters were set behind the microscope for recording. A flat-response X-ray detector (F-XRD) was set at $42\,^{\circ }$ relative to the hohlraum axis to measure the radiation temperature. A neutron time-of-flight (NTOF) spectrometer was set at the equator of the target chamber to detect the neutron yield. A pinhole camera was used to monitor the laser entrance situation. During the experiment, 48 frequency-tripled laser beams ($\lambda$ = 0.35 $\upmu$m, 1890 J in 2.1 ns per beam) irradiated the hohlraum wall at four different angles ($55\,^{\circ }$, $49.5\,^{\circ }$, $35\,^{\circ }$, $28.5\,^{\circ }$) relative to the hohlraum axis, from two symmetrically positioned 1.2-mm-diameter laser entrance holes.

From the calibration of the linear response range of the X-ray IP^21^, we could obtain the scaling coefficients between the experimental photo-stimulated luminescence (PSL) and the number of counts in a high-purity germanium detector (HPGe), which was used to calibrate the IP. Simultaneously, by calibrating the intrinsic peak efficiency of the HPGe^22^, we obtained the ratio of the counts of the HPGe detector to the number of irradiating monoenergetic photons. Thus, combining the scaling coefficient with the ratio^21,22^, when an 8-keV X-ray photon irradiated the IP, the PSL response value recorded by the IP was $\eta _1 = 1.75 mPSL / \gamma _{8 keV}$. In addition, the filters used in this experiment were 40 $\upmu$m Al + 120 $\upmu$m Be, with a transmissivity of $T = 53.09\%$ according to the experimental measurements and 56.63% according to the calculations. As a result, the *n* value could be obtained by scanning the PSL value distribution *P* (unit: *mPSL*) on the IP, i.e.,

3 $$\begin{aligned} {} n = \frac{P}{\eta _1 T}. \end{aligned}$$

Thus,

4 $$\begin{aligned} {} N = \frac{4 \pi P}{\eta _1 T \eta }. \end{aligned}$$

**Figure 4:**
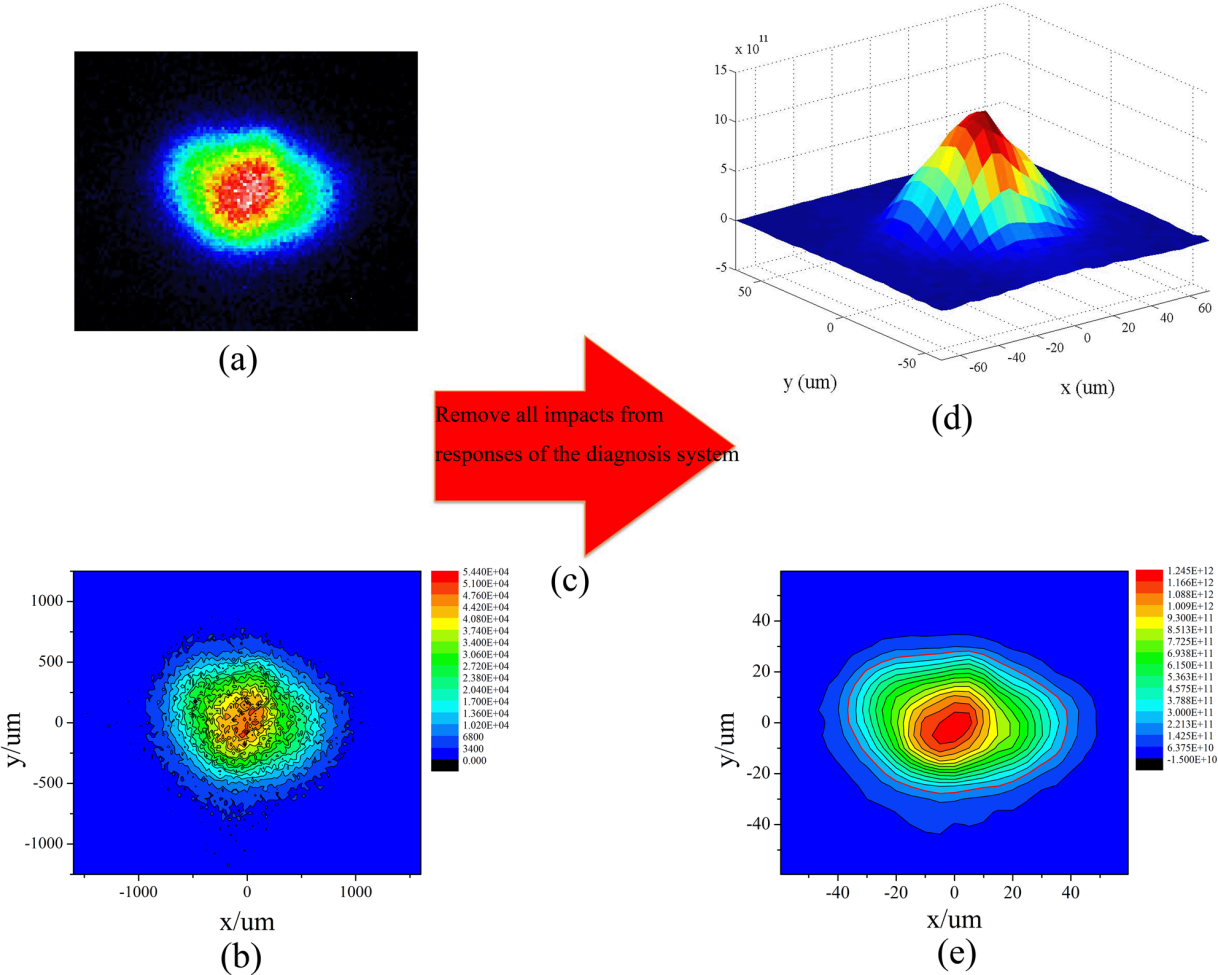
(Color online) (**a**) Observation results of imaging plate—the original image. (**b**) Original image coupled with the contour line. (**c**) Procedure of quantitative data process. (**d**) Photon number distribution images of quantitative and 5-$\upmu$m-space-resolving 8 keV self-emission of hotspot. (**e**) Photon number distribution images coupled with the contour line. The red line is the 17.81% contour.

**Figure 5:**
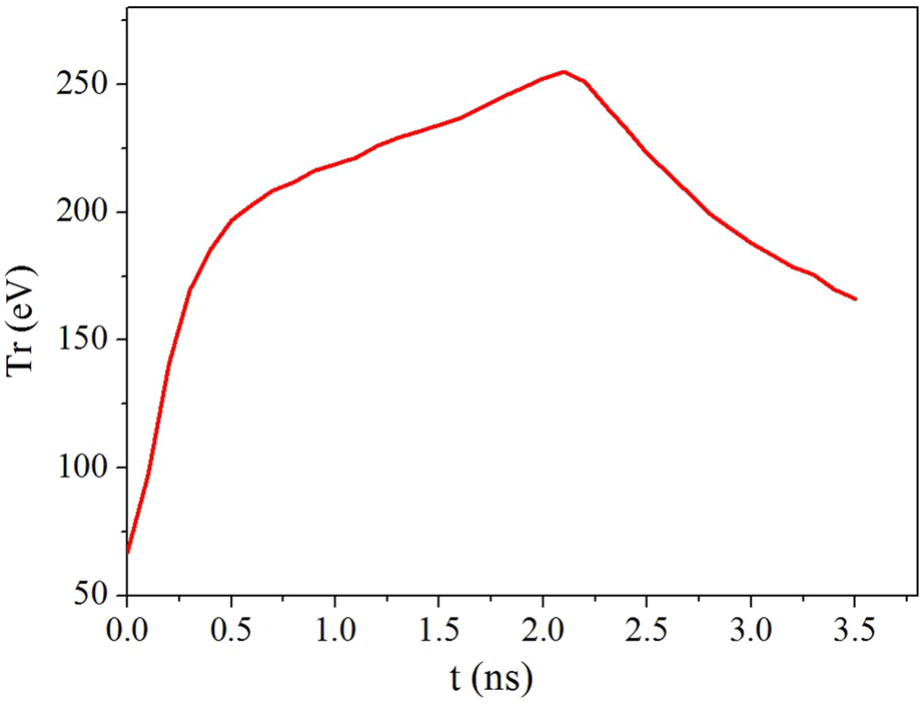
(Color online) Detected hohlraum radiation temperature curve by F-XRD.

Figure 4(a) shows the observation results of the IP of the best channel in the eight-image KB microscope. The contour line was used for characterizing the hotspot self-emission intensity in Fig. 4(b). This result is typical of most studies that use a KB microscope to observe an implosion hotspot^8–11^. However, Fig. 4(b) reveals that the hotspot emission intensity is mixed and disordered and it is difficult to extract the distribution characteristic information. Most importantly, in this work, we removed the influences of the IP background, IP responses, filter transmission, and spatial responses of the KB microscope, as shown in Fig. 4(c). Figure 4(d) and (e) show the photon number distribution images of the quantitative and 5-$\upmu$m-space-resolving 8 keV self-emission of the hotspot. They show the substantive, ordered, and clear characteristics of the hotspot self-emission. The distribution has a peak in the center and is flat at the ends, which corresponds to the electron temperature distribution of the hotspot^2,23^. This reveals that the electron temperature, rather than the density, dominates the hotspot self-emission. Interestingly, the center (< 20 $\upmu$m) profile differs from the outer profile in the hotspot from Fig. 4(e). This indicates that there is a marked P2 drive asymmetry in the capsule implosion compression. Even though this asymmetry does not affect the whole emission characteristic of the hotspot (peak in the center and flat at the ends), it may heavily affect the energy coupling efficiency of the capsule implosion. We discuss this in the following section. The maximum value of the 5 $\upmu$m $\times$ 5 $\upmu$m mesh in the photon number distribution of the hotspot is 1.24 $\times$ $10^{12}$, and the total photon number is 9.98 $\times$ $10^{13}$, corresponding to 1.28 $\times$ $10^{-1}$ J energy. Additionally, the radiation temperature measured by F-XRD in the hohlraum is shown in Fig. 5 with a peak value of approximately 250 eV. The neutron yield detected by the NTOF spectrometer reaches 3.6 $\times$ $10^{10}$.

## Simulation and analysis

We used the LARED-S code^24,25^ to conduct one-dimensional simulations of this experiment, for comparison with the experimental results. LARED-S is a multi-material radiation hydrodynamic code including the second-order Godunov scheme with the material interface tracker based on the volume fraction method, flux-limited electron and ion heat conduction, multigroup diffusion transport of charged particles, and multigroup diffusion radiation transport. In addition, the real equation of state and tabular database of radiation parameters were incorporated into this code. In the simulation performed in this study, the radiation temperature driving the capsule was selected as the temperature measured by the F-XRD set at $42\,^{\circ }$ relative to the hohlraum axis, as shown in Fig. 5.

To calculate the number of monochromatic X-ray photons produced by the hotspot, we implemented the following methods. The state distribution parameters of the hotspot were obtained through numerical simulation. The high-energy X-rays emitted by the hotspot were mainly due to bremsstrahlung emission^26^, and the bremsstrahlung spectral energy emission per unit mass (unit: *W*/*g*) was^26^

5 $$\begin{aligned} {} B_\nu = \frac{16\pi }{3\sqrt{6\pi }} \frac{e^6}{m_e^{\frac{3}{2}}c^3m_p^2} \frac{z^3\rho }{(kT_e)^{\frac{1}{2}}A^2} e^{-\frac{h\nu }{kT_e}}, \end{aligned}$$

where *e* (unit: *C*) is the elementary charge, *z* is the degree of ionization, $\rho$ (unit: $g/cm^3$) is the density, *h* (unit: $J \cdot s$) is the Planck constant, $\nu$ is the frequency (unit: $s^{-1}$), $m_e$ (unit: *g*) is the electron mass, *c* (unit: *m*/*s*) is the velocity of light, $m_p$ (unit: *g*) is the proton atomic mass, *k* (unit: *J*/*keV*) is the Boltzmann constant, $T_e$ (unit: *keV*) is the electron temperature, and *A* is the atomic weight. For photons of frequency $\nu \pm \Delta \nu$, the bremsstrahlung power per unit mass was^26^

6 $$\begin{aligned} p_{br}^{\nu , \Delta \nu }&= 4\pi \int _{\nu -\Delta \nu }^{\nu +\Delta \nu } B_\nu d\nu \end{aligned}$$

7 $$\begin{aligned}&= \frac{32\pi }{3\sqrt{6\pi }} \frac{e^6}{m_e^{\frac{3}{2}}c^3 \hbar m_p^2}\frac{z^3\rho (kT_e)^{\frac{1}{2}}}{A^2}\int _{x-\Delta x}^{x+\Delta x}e^{-x}dx \end{aligned}$$

8 $$\begin{aligned}&= 1.74\times 10^{17}\frac{z^3\rho (T_e)^{\frac{1}{2}}}{A^2}(e^{-(x-\Delta x)}-e^{-(x+\Delta x)}), \end{aligned}$$

where the units of $p_{br}^{\nu , \Delta \nu }$ are *W*/*g*, $x=\frac{h\nu }{kT_e}$, and $\Delta x=\frac{h\Delta \nu }{kT_e}$. In this work, $\nu$ corresponds to 8 *keV*, and $\Delta \nu$ corresponds to 0.39 *keV*.

**Figure 6:**
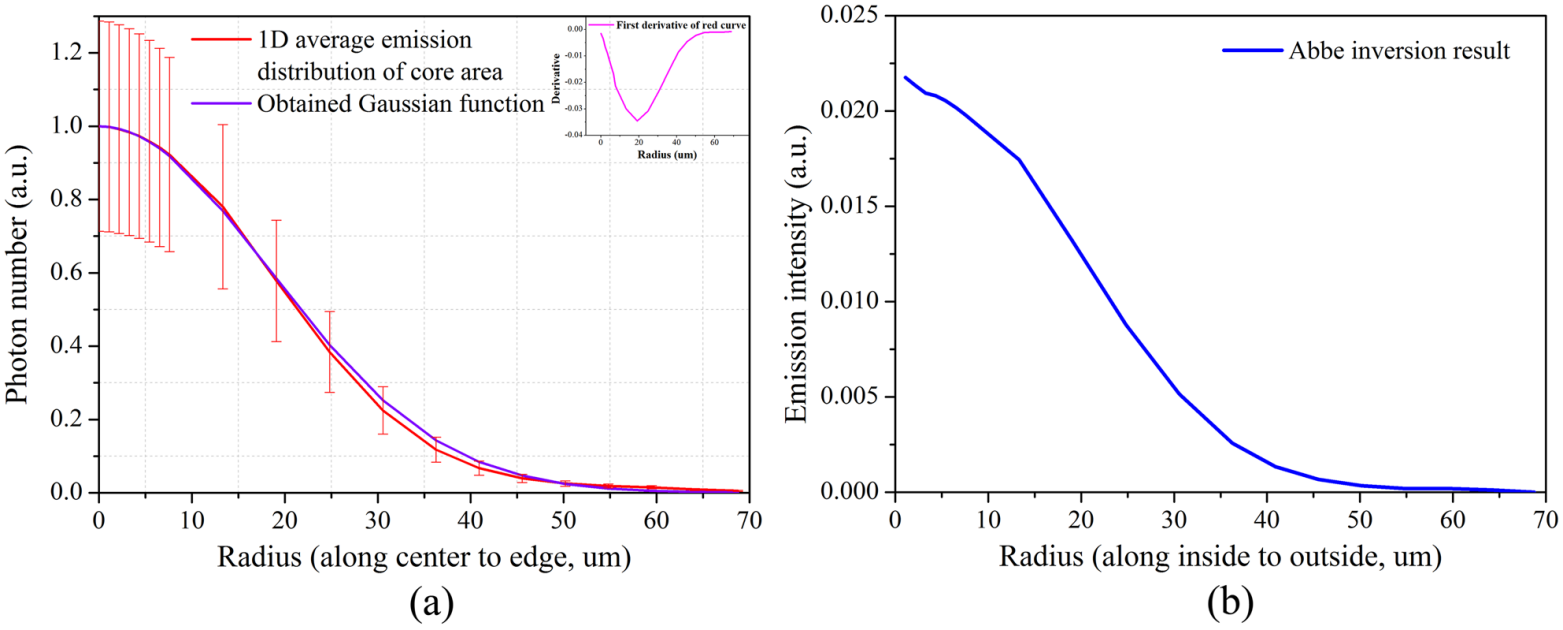
(Color online) (**a**) One-dimensional average distribution of the core area emission extracted from Fig. 4(d) (red curve). Purple curve is the obtained Gaussian function curve. Upper right curve is the first derivative of the 1D average distributions. (**b**) Abbe inversion results of the core emission area.

Through the one-dimensional theory simulation without drive asymmetry, the number of 8 ± 0.39-keV X-ray photons produced in the core area of the implosion capsule was obtained as approximately 2.18 $\times$ $10^{14}$. The DD gas fuel area created 1.03 $\times$ $10^{14}$ 8 ± 0.39-keV X-ray photons, namely the emission of the hotspot. Thus, the photons produced by the gas fuel were only 47% of the emission of the entire core area, which includes the DD hotspot and the capsule ablator. According to the calculation, approximately 53% of the photons emerged in the capsule ablator with a thickness of approximately only 1 $\upmu$m. For a comparison with the experimental results, we extracted the one-dimensional average distribution of the core area emission from Fig. 4(d) by using the interpolation method as shown in Fig. 6(a). Based on the average distribution, we obtained the Abbe inversion of the experimental results as shown in Fig. 6(b). The profile produced by the Abbe inversion is the photon number distribution along a radius from the inside to the outside of the three-dimensional hotspot. According to the theoretical simulation, the 53% emission would show a bump at a large radius, with thickness 1 $\upmu$m in Fig. 6(b) but not in Fig. 6(a). This is because the bump at a large radius could be submerged in the integrated photon number distribution (Fig. 6(a)) with a relatively short integrated path. However, there was no strong emission corresponding to the 53% emission. The calculated neutron yield of 6.9 $\times$ $10^{10}$ is almost twice as much as the experimental result. Thus, the following two-dimensional calculation took the P2 drive asymmetry and reduced ablator emission into consideration.

**Figure 7:**
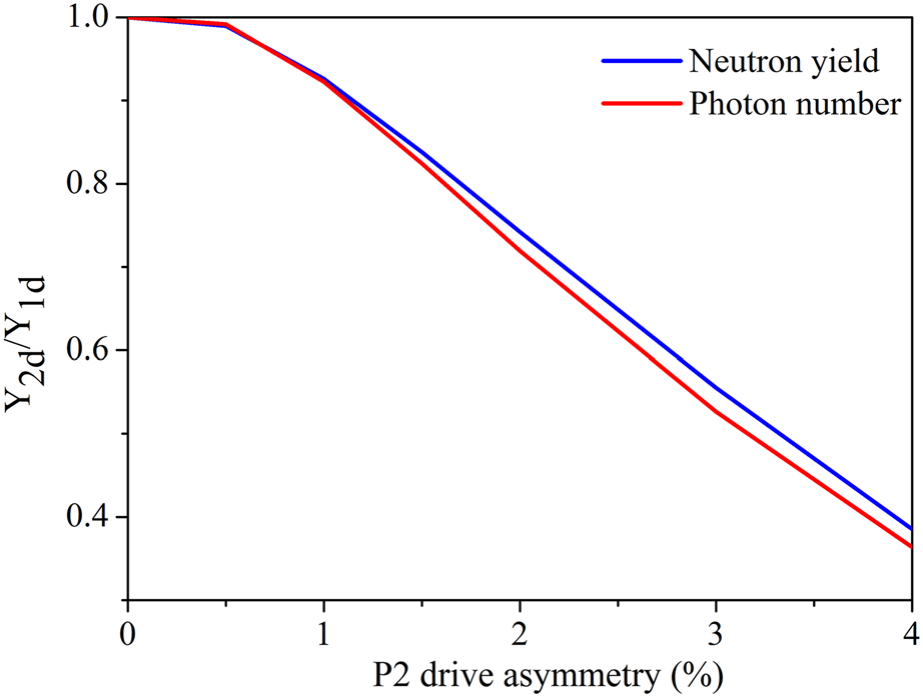
(Color online) Horizontal axis is P2 drive asymmetry of the radiation source on the capsule. Vertical axis is the ratio of the 2D calculation results to the 1D calculation results. Black curve shows the DD-fusion neutron yield variation according to the P2 drive asymmetry, and red curve shows number of 8 ± 0.39-keV photons emitted by hotspot according to P2 drive asymmetry.

Two-dimensional simulations were conducted by varying the P2 symmetry of the radiation drive, and the influence on the implosion hotspot distortion was analyzed. Figure 7 shows the variation in the DD-fusion neutron yield and 8 ± 0.39-keV photons emitted by the hotspot according to the P2 drive asymmetry. This asymmetry originates 100 $\upmu$m away from the ablating plane and it is the average value of the time taken by the radiation source added to that of the maximum implosion velocity reached. Figure 7 indicates that the P2 drive asymmetry is closely related to the fusion neutron and the hotspot self-emission photon. The distortion of the hotspot will lead to an obvious decrease in the neutron yield and self-emission photon number.

**Figure 8:**
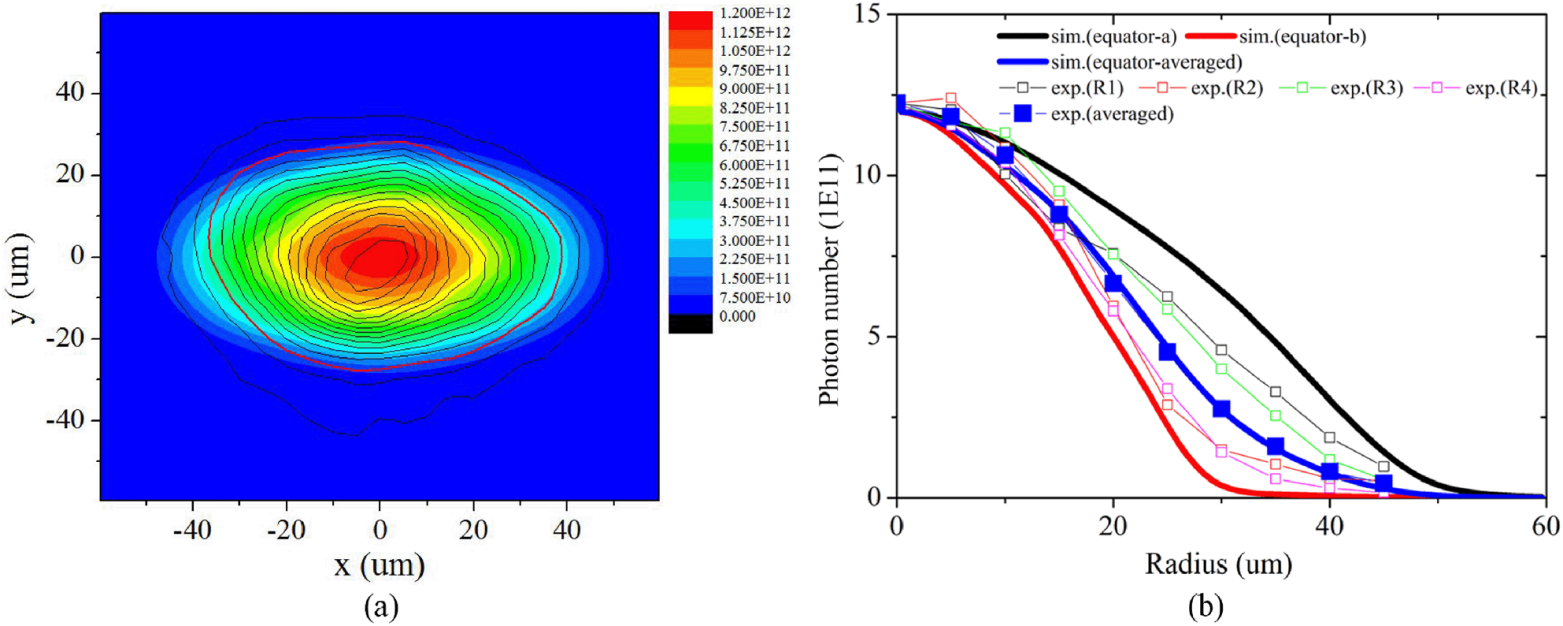
(Color online) (**a**) Self-emission distributions of the core area including DD fuel and self-emission of ablator with the P2 drive asymmetry=2.5% as per calculations. The contour lines are extracted from the experimental results in Fig. 4(e). (**b**) Comparison of radial distributions of the number of photons extracted according to experimental and simulation results, namely Figs. 4(e) and 8(a) respectively. Black solid curve shows distribution along the long axis equator-a of the simulation results, red solid curve is the distribution along the short axis equator-b, and blue solid curve is the average distribution along different angles. The squares are experimental data from Fig. 4(e). Hollow squares are the radial distributions of different radii along up, down, left, and right orientations (R1 to R4). Blue solid squares are the average results of eight radii, including another four radii $45\,^{\circ }$ relative to R1–R4. The center of all the radii is the point of maximum emission intensity.

By fitting the experimental results shown in Fig. 4(e), we have the P2 radiation drive asymmetry=2.5%, and the capsule ablator emission decreased to 0.3 times the DD hotspot value. Considering the heavy hotspot mix^27,28^ possibly existing at the interface, the decrease in the ablator emission should be reasonable. Even though the drive asymmetry was relatively large (2.5%), the shape of the core emission area including hotspot and ablator will not be too flat. Figure 8(a) shows the two-dimensional simulation results of the core emission area. For comparison, the contour lines of the experimental results were added. Figure 8(b) shows the distribution of the number of photons in the experiments and simulations along the emission area radius for comparison. At the center of the emission area (< 20 $\upmu$m), the deviation between the experimental and simulation results was less than 3%. In the core emission area ($\ge$ 20 $\upmu$m), all the experimental data lie between the long axis equator-a and short axis equator-b distribution curves of the simulation data. The data dispersal of both experimental and simulation results in this area indicate the obvious P2 drive asymmetry. The average values of the experimental and simulation results are highly congruent (the blue solid squares and the blue solid line). This is one of the two main aspects for the simulations to be matched to the experimental data. Especially in the center 20-$\upmu$m-radius distributions, there is hardly any hotspot mix considering that the main mechanism for the mix is dominated by the interface mix. The total photon number and neutron yield in the simulation were 9.3 $\times$ $10^{13}$ and 4.5 $\times$ $10^{10}$, respectively, and the experimental results were 9.98 $\times$ $10^{13}$ and 3.6 $\times$ $10^{10}$, respectively. The total photon number is the other main aspect for the simulations to be matched to the experimental data. We can see that the simulations almost reproduce the experimental observations, i.e., the profile of monochromatic photon emission image, distributions and total number of monochromatic photons, and neutron yield. However, there were some differences between the 2D simulation and the experimental observations, indicating the limitation of the 2D code on 3D implosion simulation. As expected, it is difficult to simulate the hotspot mix, which is slight and complicated.

**Figure 9:**
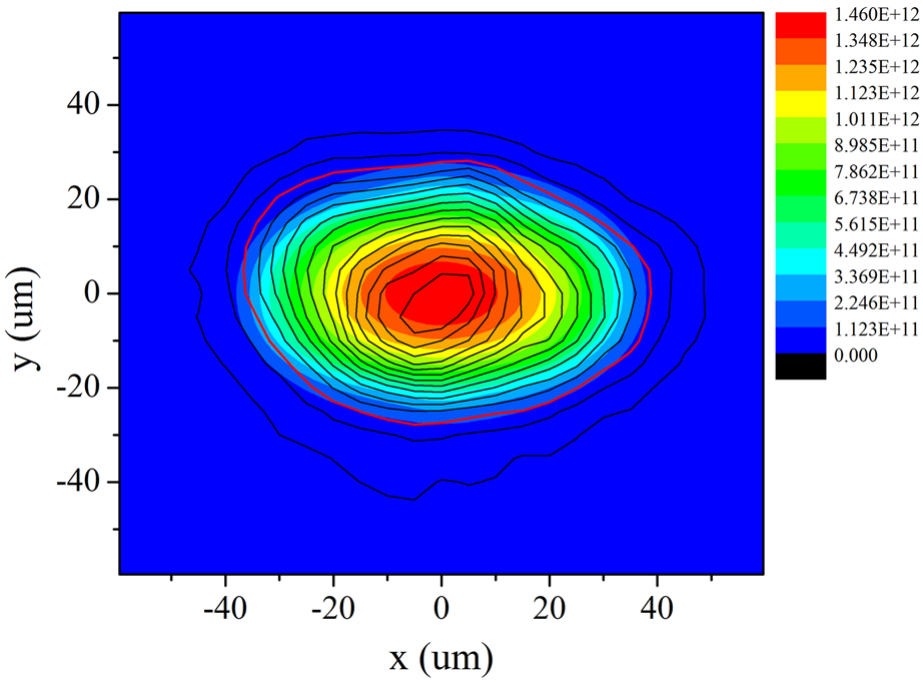
(Color online) Self emission distributions of DD fuel hotspot only. The contour lines are extracted from the experimental results of Fig. 4(e).

On removing the ablator emission in the simulation and comparing it with the experimental results, shown in Fig. 9, the profile of the gas fuel hotspot was found to be highly congruent with the contour line—17.81% level of the experimental peak emission. The 17.81% contour also agrees with the 17% contour in many other works^29,30^. Although the ablator emission is 0.3 times in that contour range, the hotspot emission dominates, especially at the center. The hotspot has a 37.60 $\upmu$m long axis and a 28.00 $\upmu$m short axis by the 17.81% contour. Thus, the average radius is approximately 32.80 $\upmu$m, and the contraction ratio $C_r$ of the implosion is approximately 13.72, indicating that $C_r$ should be promoted by two to three times for ignition based on the 100 kJ laser facility.

By analyzing the experimental results in Figs. 4(d) and 6(a), we think the 1D distributions of the core emission are consistent with the Gaussian function

9 $$\begin{aligned} f(r)=a e^{-\frac{(r-b)^2}{2c^2}}, \end{aligned}$$

where *r* is the radius of the core emission area (in $\upmu$m); *a* is the maximum emission intensity, which is one with intensity normalization; *b* is the radius of the maximum emission intensity, namely zero; and *c* is the standard deviation. When $f(r)=17.81\%$, $r=32.80$ and $r/c=1.86$; thus, $c=17.63$. For contrast, the first derivative of the 1D average distributions of the core area emission is also shown in Fig. 6(a). The extreme value of the first derivative is at $r=19.07$, which should also be the value of *c*. The difference between 19.07 $\upmu$m and 17.63 $\upmu$m is only 1.44 $\upmu$m. Therefore, we chose the average value of 18.35 as *c*,

10 $$\begin{aligned} f(r)=e^{-\frac{r^2}{2\times 18.35^2}}. \end{aligned}$$

As shown in Fig. 6(a), the curve of the Gaussian function is highly consistent with the 1D experimental distributions, indicating the accurate Gaussian characteristics of the core emission. This enhances our basic knowledge of the hotspot area.

Finally, we discuss the uncertainties in the hotspot self-emission observation experiments, which may arise from four sources. The first is generated by the response coefficient of the X-ray IP $\eta _1$ and is approximately 10.30%^21,22^. The second stems from the influence of the microscope mispointing the $\eta$ values and is less than 26.47%. The third is derived from attenuation of the PSL value distribution *P* on the IP and is approximately 4%. The fourth arises during the experimental calibration of the filter *T* and is 0.5%. Thus, the total relative uncertainties in the hotspot self-emission observation experiments performed in this study were 28.69%.

## Conclusions

In conclusion, the 8 ± 0.39-keV monochromatic X-rays emitted from a hotspot were quantitatively observed in 5-$\upmu$m spatial resolution using an eight-image KB microscope and, for the first time, after removing the influences of the diagnosis system responses. This was achieved in implosion experiments performed at the 100 kJ laser facility in China. The quantitative results extend certain aspects of our knowledge of the hotspot, such as the quantitative monochromatic photon number distribution, the profile of the hotspot emission area, and total energy. The 2D simulation results are highly consistent with the experimental results, i.e., the profile of the monochromatic photon emission image, the distribution and total monochromatic photon number, and the neutron yield. We found that the P2 drive asymmetry=2.5% and the capsule ablator emission decreases to 0.3 times, owing to drive asymmetry and the hotspot mix. In addition, the origins of the 17.81% contour profile of the deuterium-deuterium hotspot and the accurate Gaussian source approximation of the core emission area in the implosion capsule are clarified in detail.

This quantitative investigation will be very useful in validating the basic quantitative physical understanding of the stagnation conditions of implosion compression in ICF research. Further, these significant improvements provide precise parameters and corrections for future simulations of hohlraum energetics and capsule implosion, and promote the development of related energetics and implosion physics research in ICF. In the future, more extensive work will focus on the measurement of X-rays emitted from the hotspot at different energy points, which could provide quantitative and high-spatial-resolution observations of the distributions corresponding to the hotspot geometric structure, emission intensity, 2D electron temperature ($T_{e} = \frac{h(\nu _{2}-\nu _{1})}{k_{B}lg(I_{1}/I_{2})}$ , where $\nu _{1}$ and $\nu _{2}$ are the frequencies of two monochromatic X-rays emitted from the hotspot, $I_{1}$ and $I_{2}$ are the intensity distributions of the two monochromatic X-ray images, and *h* and $k_{B}$ are the Planck constant and Stefan-Boltzmann constant respectively), density, and internal energy^31^.
